# A Low-Noise X-ray Astronomical Silicon-On-Insulator Pixel Detector Using a Pinned Depleted Diode Structure

**DOI:** 10.3390/s18010027

**Published:** 2017-12-23

**Authors:** Hiroki Kamehama, Shoji Kawahito, Sumeet Shrestha, Syunta Nakanishi, Keita Yasutomi, Ayaki Takeda, Takeshi Go Tsuru, Yasuo Arai

**Affiliations:** 1 Information and Communication Systems Engineering, National Institute of Technology, Okinawa College, Okinawa 905-2171, Japan; hkame@okinawa-ct.ac.jp; 2 Research Institute of Electronics, Shizuoka University, Shizuoka 432-8011, Japan; sumeet@idl.rie.shizuoka.ac.jp (S.S.); snakani@idl.rie.shizuoka.ac.jp (S.N.); kyasu@idl.rie.shizuoka.ac.jp (K.Y.); 3 Department of Applied Physics and Electronic Engineering, University of Miyazaki, Miyazaki 889-2192, Japan; takeda@astro.miyazaki-u.ac.jp; 4 Department of Physics, Kyoto University, Kyoto 606-8502, Japan; tsuru@cr.scphys.kyoto-u.ac.jp; 5 High Energy Accelerator Research Organization, Tsukuba, Ibaraki 305-0801, Japan; yasuo.arai@kek.jp

**Keywords:** SOI X-ray detector, high energy resolution, low noise, back-side surface potential pinning structure

## Abstract

This paper presents a novel full-depletion Si X-ray detector based on silicon-on-insulator pixel (SOIPIX) technology using a pinned depleted diode structure, named the SOIPIX-PDD. The SOIPIX-PDD greatly reduces stray capacitance at the charge sensing node, the dark current of the detector, and capacitive coupling between the sensing node and SOI circuits. These features of the SOIPIX-PDD lead to low read noise, resulting high X-ray energy resolution and stable operation of the pixel. The back-gate surface pinning structure using neutralized p-well at the back-gate surface and depleted n-well underneath the p-well for all the pixel area other than the charge sensing node is also essential for preventing hole injection from the p-well by making the potential barrier to hole, reducing dark current from the Si-SiO_2_ interface and creating lateral drift field to gather signal electrons in the pixel area into the small charge sensing node. A prototype chip using 0.2 μm SOI technology shows very low readout noise of 11.0 e^−^_rms_, low dark current density of 56 pA/cm^2^ at −35 °C and the energy resolution of 200 eV(FWHM) at 5.9 keV and 280 eV (FWHM) at 13.95 keV.

## 1. Introduction

X-ray astronomical satellites require low-noise high-time-resolution high-spatial-resolution detectors. X-ray charge-coupled devices (CCDs) are currently used as the standard imaging devices because they offer Fano-limited spectroscopic performance (~120 eV in FWHM at 6 keV) with a low readout noise of about 3 e^−^_rms_ [1,2]. However, X-ray CCDs suffers from the poor time resolution (a few seconds) and the low dynamic range (0.3–10 keV) [3]. To meet the requirements of both low noise and high time-resolution, complementary metal oxide semiconductor (CMOS)-based event-driven type of detectors are being developed. One of techniques to implement CMOS event-driven detectors is to use a hybrid detector structure which uses stacking of a silicon detector and CMOS readout electronics through Indium bump interconnections [4]. However, hybrid CMOS detectors have a limitation on large pixel number, small pixel size, and high production yield. Another technique for CMOS event-driven detectors is to use monolithic CMOS detector technology. Monolithic CMOS detectors do not require any mechanical bump bonding between detector and readout circuits. Pixel size can be relatively small for higher spatial resolution. The silicon-on-insulator pixel (SOIPIX) detector technology being developed by the High Energy Accelerator Research Organization (KEK, Ibaraki, Japan) and Lapis Semiconductor, Inc. (Kanagawa, Japan) is a good platform for implementing a monolithic CMOS event-driven detector [3]. The SOIPIX is an active pixel sensor based on a semiconductor pixel detector realized with a CMOS fully depleted (FD-) SOI technology. 

**Figure 1:**
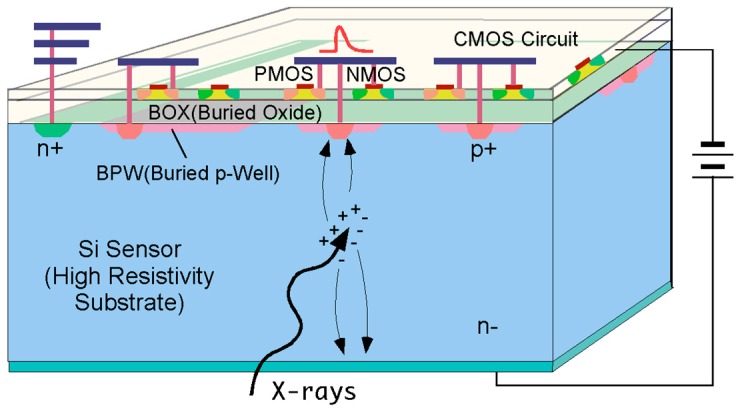
The cross-sectional view of the SOIPIX.

Figure 1 shows the cross-sectional view of the SOIPIX. The SOI wafer is composed of a thick, high-resistivity substrate for the sensing part and a thin Si layer for CMOS circuits sandwiched by a buried oxide (BOX) layer. The SOIPIX utilizes a buried p-well (BPW) as a sensing part to detect X-rays [5]. Using the 0.2 μm CMOS fully depleted (FD) SOI technology, we have been developing an event-driven X-ray pixel (XRPIX) series [6,7,8,9]. Though their basic characteristics have been gradually improved, there still are issues on the improvements of noise, charge correction efficiency, and the resulting X-ray energy resolution. The latest version of XRPIX called XRPIX3b uses a charge-sensitive amplifier (CSA) circuit in each pixel to increase the conversion gain and reduce readout noise. The XRPIX3b has achieved the readout noise of 35 e^−^_rms_ and the energy resolution of 320 eV (FWHM) at 6 keV [9]. The detector structure used for the XRPIX3b, however, has a difficulty of reduction of the sensing-node capacitance, a problem of crosstalk between the sensing node and SOI CMOS circuits, and dark current generation at the Si-SiO_2_ interface under the BOX. To solve the crosstalk problem, an SOIPIX technology using the nested-well structure has been proposed [10]. Though this technique is effective for reducing the crosstalk problem, the nested-well structure still has an issue on the large capacitance at the sensing node. The SOIPIX using double SOI layers recently reported is an attractive device for realizing a low-noise detector that exploits the merits of the middle Si layer (middle SOI) for reducing the crosstalk by shielding the sensing node from the SOI circuits and reducing the sensing-node capacitance with the structure of the depleted substrate Si surface (Si-SiO_2_ interface under the BOX layer) [11]. However, it still has issues on dark current generation at the depleted substrate Si surface, and a possibility of signal charge loss by the traps at the Si surface, leading to the degraded charge collection efficiency. 

In order to realize an X-ray pixelated detector with high energy resolution based on the SOIPIX technology, this paper proposes a novel SOIPIX using a pinned depleted diode structure. This pixel technology called the SOIPIX-PDD allows us to solve the issues of conventional SOIPIXs associated with the readout noise, dark current, crosstalk, and charge collection efficiency, while having a feature of fully depleted thick sensing region of the handle substrate which is commonly required for high energy imaging [12,13,14]. Thanks to the pinned depleted diode structure having features of the pinned Si surface layer which also acts as an electro-static shielding layer and depleted buried channel for carrier collection to the small-capacitance charge sensing node, dark current at substrate Si surface, readout noise and crosstalk are greatly reduced. This structure is also effective for high charge collection efficiency and high-speed response because the signal carriers collected run in the buried channel with the help of lateral electro-static field but without touching to the Si surface. The rest of this paper describes the pixel device structure, pixel circuits, implementation and evaluation results of the SOIPIX-PDD, and finally conclusions. 

## 2. SOI Pixel Detector Using a Pinned Depleted Diode Structure 

### 2.1. Sensor Structure Implemented on High-Resistivity Substrate

In the conventional SOIPIX as shown in Figure 1, the charge sensing node is made with a BPW at the back-gate surface of the high-resistivity Si substrate and the other part of back-gate surface is depleted. This depleted back-gate surface leads to a large dark current and charge loss due to the interface states. To increase the charge collection efficiency, the size of detector BPW must be increased and the resulting capacitance of the detector is increased, leading to a large capacitance of the sensing node. The capacitive coupling between the SOI circuits and the buried BPW may cause an additional noise and offset. To reduce the capacitive coupling between the sensing node (BPW) and SOI circuits, a nested-well structure based on the SOIPIX has been proposed [10,15]. However, the nested-well structure uses a large-size sensing plate made with neutralized BPW created underneath a buried n-well (BNW) and the capacitance of the sensing node becomes pretty large.

**Figure 2:**
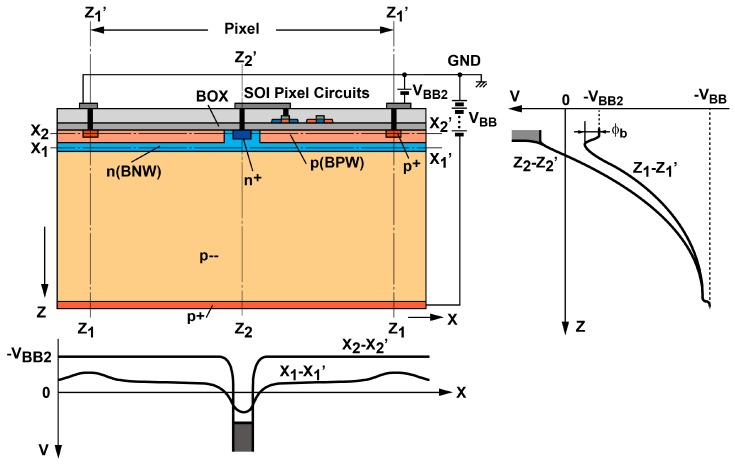
Cross-sectional view of the SOIPIX-PDD.

The SOIPIX using the pinned depleted diode structure, the SOIPIX-PDD, is developed to improve the detector performance compared with the conventional SOI pixel detector while maintaining the fundamental merit of the SOIPIX [16]. Figure 2 shows the cross-sectional view of the SOIPIX-PDD. For X-ray imaging, high negative voltage is applied at the backside of the detector for attaining a fully depleted thick substrate. A BPW is formed on the backside of the BOX for pinning the back-gate voltage of the SOI transistors to a fixed bias of V_BB2_. The BPW acts as a shielding layer between the charge sensing node and the SOI circuits, preventing the extra noise and offset generation by the coupling. 

The sufficiently highly-doped BPW as a neutral region is effective for reducing the dark current generation at Si-SiO_2_ interface under the BOX, because it works like a pinned photodiode in CCD or CMOS image sensors [17]. A BNW formed under the BPW is depleted and this layer acts as a buried channel to gather carriers generated in the pixel into the sensing node (n+) and to improve charge collection efficiency, because lateral electric field is created in this channel as shown in the potential profile of X_1_-X_1_’ and a problem of the carrier trapping at Si-SiO_2_ interface under the BOX in the conventional SOIPIX is solved by the buried channel structure. One important design issue is to minimize the leakage current from the BPW to the back-side p+ layer by creating a sufficient potential barrier φ_b_ to holes as shown in Figure 2.

**Figure 3:**
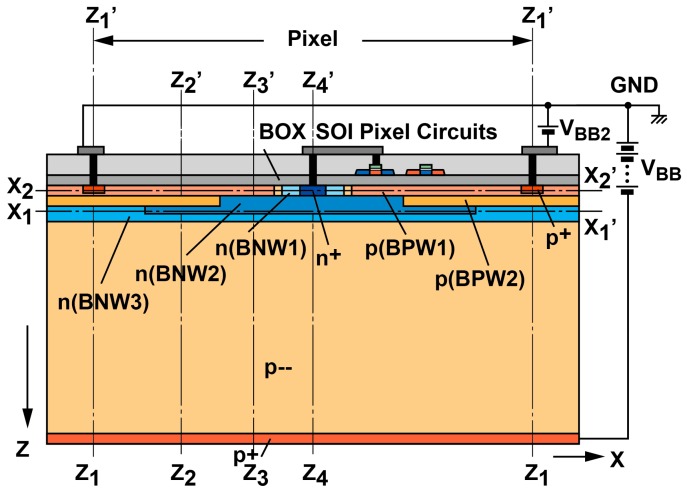
Cross-sectional view of the SOIPIX-PDD with multiple buried wells.

The SOIPIX-PDD shown in Figure 2 uses a single BNW for creating a channel with lateral electric field. If the pixel size is very large, the electric field created by this BNW only may not be sufficient for gathering charges within a time to meet the required X-ray photon incidental rate of >1 MHz. In order to create a sufficiently large lateral electric field in whole detector volume, a multiple buried-well structure for the PDD as shown in Figure 3 is used. In this detector, the lateral electric field in the channel is created by two buried p-well (BPW1 and BPW2) and three buried n-well (BNW1, BNW2 and BNW3) are used. Since the BNW3, BNW2 and BNW1 under the BPW are depleted, the charge sensing capacitance of the detector is only due to the PN junction between the BPW and n+ and a part of the BNW near n+, a high charge-to-voltage conversion gain and the resulting low readout noise are realized.

### 2.2. Simulation of Potential Profiles of the Designed SOIPIX-PDD 

Based on the pinned depleted diode with the multiple buried wells, a pixel detector for X-ray energy spectrum measurements is designed and its potential profiles are simulated by a device simulator SPECTRA. 

**Figure 4:**
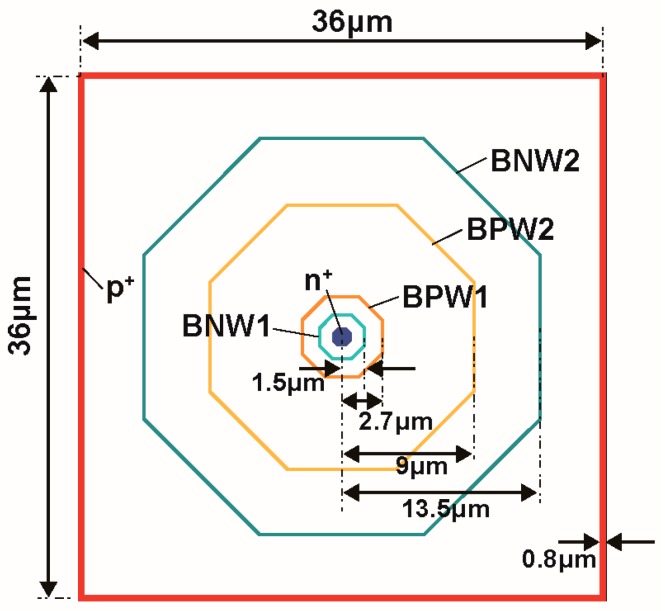
Sensor-layer patterns and dimensions of buried n-/p-wells.

Figure 4 shows the pixel layout pattern of the detector. The pixel size of the detector is 36 μm × 36 μm. A p+ layer is formed at the boundary of the pixel to bias the BPW1 for pinning the back-gate of the SOI circuits to V_BB2_. The pattern edges of the multiple buried p-/n-wells are located at 1.5 μm for BNW1 (octagonal, positive tone), at 2.7 μm for BPW1 (octagonal, negative tone), at 9 μm for BPW2 (octagonal, negative tone), 13.5 μm for BNW2 (octagonal, positive tone). The BNW3 covers all the pixel area (36 μm × 36 μm). The thickness of the sensor layer (p-type substrate) is 200 μm. The voltages applied at the sensing node (n+), the back-gate of the SOI (p+, and BPW1), and the substrate backside p+ (V_back_) are set to 3 V, −4 V (= V_BB2_) and −15 V (= V_BB_), respectively. 

**Figure 5:**
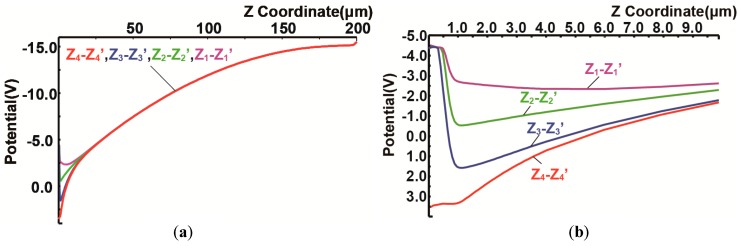
Simulated Vertical Potential Profiles, (**a**) Z = 0 to 200 μm, (**b**) Z = 0 to 10 μm.

**Figure 6:**
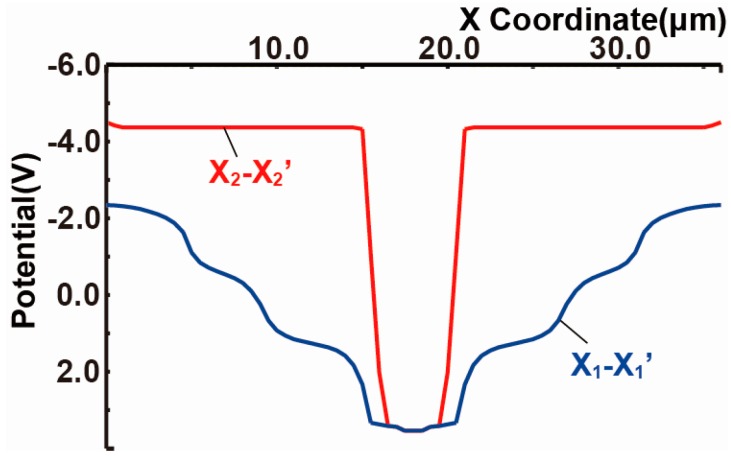
Simulated Horizontal Potential Profiles.

**Figure 7:**
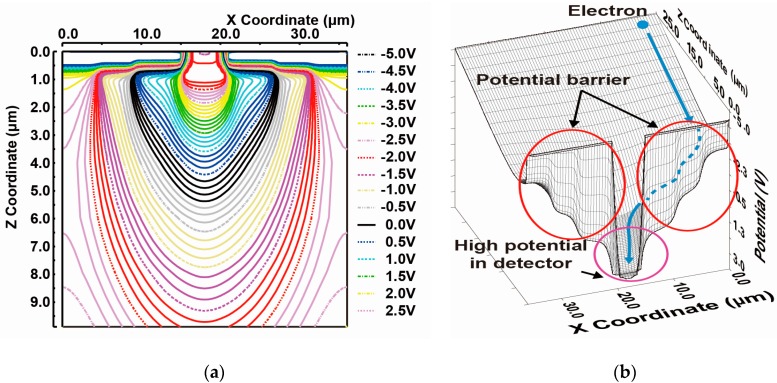
2-D (X-Z) Equipotential plot (**a**) and Bird’s Eye View of the 2-D (X-Z) Potential (**b**).

Figure 5 shows the simulated potential profiles of the designed SOIPIX-PDD with multiple buried wells. Figure 5a,b show the potential distribution of vertical cross-sections along Z_1_-Z_1_’, Z_2_-Z_2_’, Z_3_-Z_3_’ and Z_4_-Z_4_’ of Figure 3. The entire sensor layer is fully depleted from the surface to the backside. Figure 5b is a zoomed potential distribution from the depth of the surface to 10 μm. In the cross-section of Z_4_-Z_4_’, a potential profile that carriers generated in the deep inside of silicon is directly transferred to the n+ sensing node. In the cross-sections of Z_1_-Z_1_’, Z_2_-Z_2_’ and Z_3_-Z_3_’, the back-gate surface is pinned to the applied voltage (= −4 V) to the BPW1, while creating a potential barrier φ_b_ of larger than 2 V, which is sufficiently large to prevent hole injection from the BPW1. As shown in Figure 5b, the actual potential of the neutral region of the BPW1 (= −4.4 V) includes Fermi potential of −0.4 V. In the cross-sections of Z_2_-Z_2_’, Z_3_-Z_3_’ and Z_4_-Z_4_’, the carriers generated at deep inside of silicon is once coming to the near surface (buried channel) and then horizontally transferred to the n+ sensing node through the channel. Figure 6 shows the horizontal potential profiles at the cross-sections of X_1_-X_1_’ and X_2_-X_2_’. The potential profile of X_2_-X_2_’ shows the back-gate (BPW1) is pinned to −4.4 V. As shown in the potential of X_1_-X_1_’, lateral electric field is formed to collect photoelectrons in the pixel to the n+ sensing node. Figure 7 is a 2-D potential plot at near the Si substrate surface (Z = 0 to 10 µm). The simulated 2-D potential plot of the SOIPIX-PDD using multi-well structure shows that all the electrons generated from the surface to bottom of the pixel are gathered to the n+ sensing node through the depleted 3-D potential profile (X-Y-Z) of the detector, realizing high charge collection efficiency which is indicated by the shape of potential profile that collect carriers generated in the entire 3-D volume of the pixel into the n+ sensing node. 

**Figure 8:**
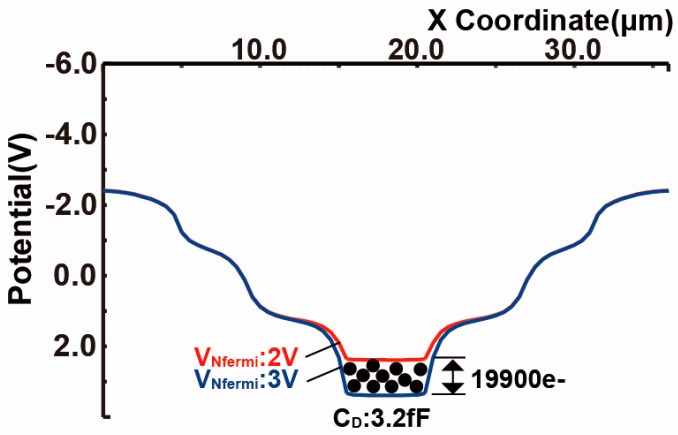
Estimation of *C_D_*.

The capacitance of the n+ sensing node denoted by *C_D_* can be estimated by the quasi-Fermi level change and the resulting change of accumulated electrons. The capacitance of *C_D_* is given by:

(1) $$C_{D}=\frac{Q_{sig}}{\Delta V_{Nfermi}}$$

where, *Q_sig_* is quasi-accumulated electrons. From the simulation results of Figure 8, *C_D_* is estimated to be *C_D_* = (*q* Δ*N_sig_*)/Δ*V_Nfermi_* = (1.602 × 10^−19^ × 19,900)/1.0 ≅ 3.2 fF. 

## 3. Charge-Sensitive Amplifier Design for Low-Noise Pixelated Detectors 

**Figure 9:**
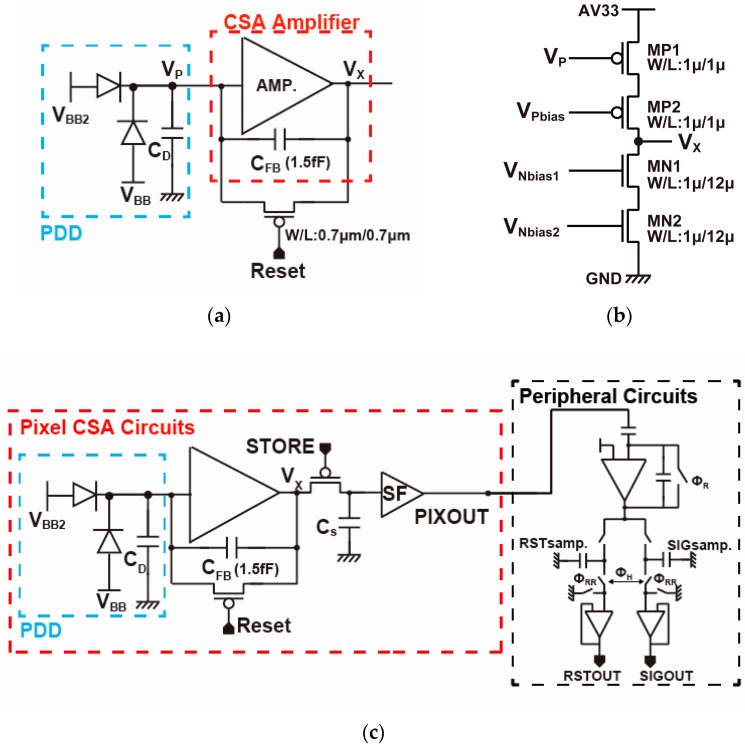
Equivalent circuits of the CSA in the pixel. (**a**) Model of CSA including those for the substrate detector, (**b**) internal amplifier, (**c**) Equivalent readout circuits chain including the pixel and peripheral.

Figure 9 shows equivalent circuits of the charge-sensitive amplifier (CSA) in the pixel including the model of the SOI substrate detector. In the SOIPIX-PDD, the SOI substrate detector is modeled with two diodes, D_1_ and D_2_, the stray capacitance *C_D_* at the n+ charge sensing node of the substrate detector. The charge-to-voltage conversion gain of the CSA is given by

(2) $$G_{C}=q\frac{G_{AMP}}{C_{D}+C_{I}+C_{FB}\cdot G_{AMP}}$$

where *C_I_* is the input capacitance of the internal amplifier, *C_FB_* is the feedback capacitance of the CSA, *G_AMP_* is the DC open-loop gain of the internal amplifier, and q is the elementary charge. If *G_AMP_* >>1, it is approximated as:

(3) $$G_{C}=\frac{q}{C_{FB}}$$

The conversion gain is solely determined by *C_FB_* with a large gain internal amplifier, and therefore a very sensitive CSA is realized if *C_FB_* is designed to be very small. 

**Figure 10:**
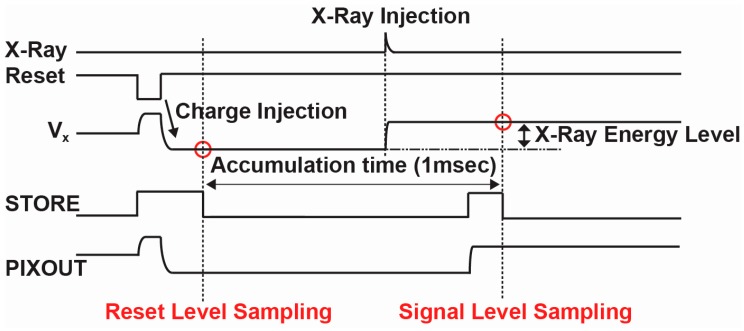
Pixel Timing Diagram.

The timing for the pixel operation is shown in Figure 10. The CSA with the pinned depleted diode detector (PDD) can be used for an event-driven type pixel using an in-pixel comparator as used in [9]. To evaluate the detector’s basic characteristics, a simple integration type of operation is used here. A PMOS reset transistor is used for better dynamic range. After the reset switch is turned off, there is a charge injection from the reset transistor. The charge injection by the reset transistor is controlled by a proper choice of the transistor size and low-level voltage applied to the reset transistor. The operating point of the amplifier is shifted to relatively low level of 0.9 V by the charge injection at the output of the amplifier. 

After the reset operation, the reset level of the CSA output is sampled at a sample-and-hold capacitor C_S_, and then the detector (and the CSA) waits an event of X-ray injection during the accumulation time shown in Figure 10. After that, the signal level of the CSA output is sampled at C_S_ again. Using a switched-capacitor CDS circuit in the peripheral circuits, the CDS (correlated double sampling) for cancelling the reset noise of the CSA can be carried out. To do this, the reset and signal levels of the CSA output sampled in the C_S_ are read out to the peripheral CDS circuit. 

**Figure 11:**
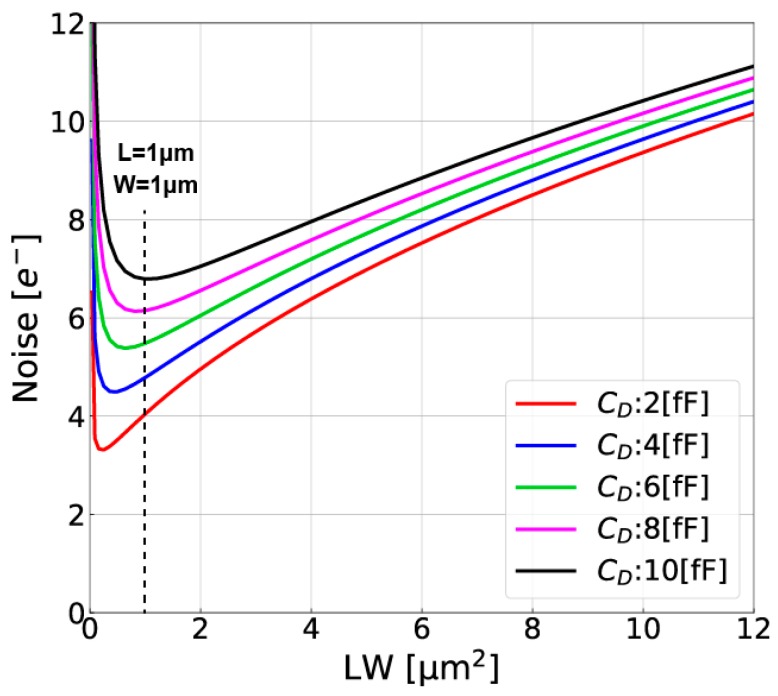
Input-referred Noise as a Function of LW of MP1 and *C_D_*.

The use of very small capacitance of *C_FB_* and the resulting high conversion gain are effective for reducing the noises superimposed after the CSA such as those of an in-pixel source follower buffer, peripheral readout circuits, output buffer amplifier, and A-to-D converter. Then, the noise of the CSA is dominated by the capacitance of the charge sensing node of the PDD detector and the design of the internal amplifier. The input-referred noise of the designed CSA shown in Figure 9 is approximately expressed as:

(4) $$N_{n}=\frac{\sqrt{2}}{G_{C}}\sqrt{\frac{1}{\beta_{F}}\frac{\xi_{A}k_{B}T}{C_{S}}+\frac{N_{f}}{\beta_{F}{}^{2}}\left(\varepsilon +\ln\frac{T_{CDS}}{\tau_{CSA}}\right)}$$

where *ξ_A_* is the excess thermal noise factor of the internal amplifier, *T_CDS_* the time difference of the two samples in the correlated double sampling operation used in the reset noise cancelling of the CSA, *N_f_* the flicker noise coefficient of the input transistor of the internal amplifier, *ε* = 0.577… Euler’s constant, *k_B_* the Boltzmann’s constant and T the absolute temperature, *β_F_* the feedback factor of the CSA given by:

(5) $$\beta_{F}=\frac{C_{FB}}{C_{FB}+C_{D}+C_{I}}$$

and *τ_CSA_* the time constant that determines the response time of the CSA which is given by

(6) $$\tau_{CSA}=\frac{C_{S}}{g_{mA}\beta_{F}}$$

where *g_mA_* is the trans-conductance of the internal amplifier [18,19]. The first and second terms in the square root of Equation (4) is due to the thermal and flicker (1/f) noises of the CSA, respectively. Equation (4) indicates that the noise is much dependent on *β_F_* or the ratio of *C_FB_* to *C_D_* + *C_I_*. For a low-noise CSA, the reduction of *C_D_* and *C_I_* is very important while using small *C_FB_* for high conversion gain. The effort for highly-sensitive substrate detector described in Section 2 reduces *C_D_*. The input capacitance of the internal amplifier *C_I_* is inversely proportional to the size (channel length (L) times channel width (W)) of input transistor (MP1 of Figure 9b). The flicker noise coefficient *N_f_* is also inversely proportional to the size (LW) of MP1 if the noises due to other transistors (MP2, MN2, and MN3) are not influenced. Therefore there exists an optimal choice of the size of MP1 to minimize the input-referred noise depending on *C_D_*, *C_FB_* and other parameters that influence Equation (4). Figure 11 shows calculated input-referred noise as a function of the transistor size of MP1 with *C_D_* as a parameter. In this calculation, *C_FB_* =1.5 [fF], T_CDS_ = 1 [ms], $\tau_{CSA}$ =0.2 μs, C*_S_* = 240 [fF] and other parameters are picked up by the PDK of the 0.2 μm SOI technology. According to the simulation results of the PDD detector, *C_D_* is estimated to be 3.2 fF. From Figure 11 and with the optimum transistor size (WL = 0.36 μm^2^), the noise level of 4.1 e^−^_rms_. is expected. However, this noise is critically increased if the flicker noise of transistors is larger than that used in this calculation. The use of larger transistor size leads to lower flicker noise and robust to noise increase. The design of the CSA for implementation uses WL = 1.0 μm^2^ for MP1 and the expected noise level is 4.5 e^−^_rms_. 

## 4. Implementation and Measurements

**Figure 12:**
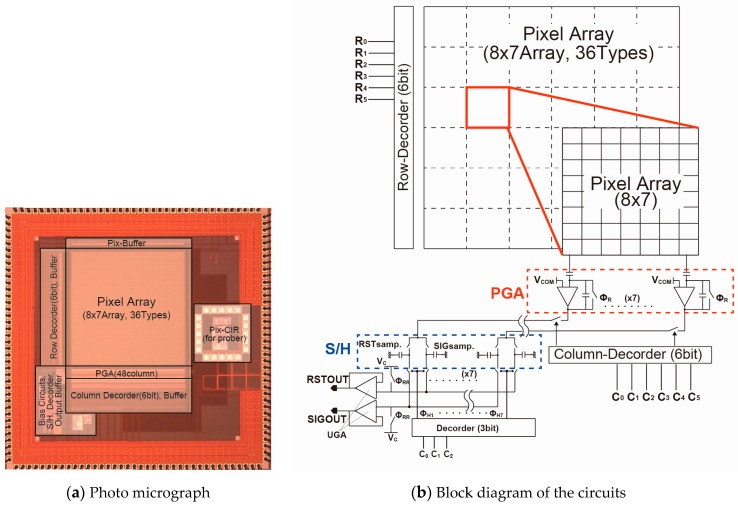
Implemented chip.

An experimental chip to evaluate the pixel performance of the SOIPIX-PDD was manufactured using 0.2 μm SOI technology as summarized in Table 1. Figure 12a shows the chip microphotograph of the sensor chip. The chip includes 6 × 6 = 36 types of pixel arrays each of which has 8 (V) × 7 (H) pixels and respective readout circuits as shown in Figure 12b. The pixel size is 36 μm × 36 μm. All the circuits and test elements are implemented in the chip die size of 4.45 mm × 4.45 mm. In the following measurement results, the 8 × 7 pixel array of a standard design whose detector dimensions and circuit parameters are described in Section 3 is used. 

### 4.1. Basic Characteristics of the SOI Pxel with Pnned Depleted Diode Structure

Basic characteristics of the proposed pixel using the SOI technology with PDD structure as the substrate detector are measured. In the following measurement results if not stated, the applied backside bias (V_BB_) is −60 V, surface-side back-gate bias (V_BB2_) is −2 V, the power supply voltage of the analog/digital pixel circuits is 3V. The pixel location of (x = 2, y = 5) if not stated is picked up for the pixel characterization because the average noise level is obtained at this pixel as shown in Figure 17 below.

The implemented pixel has event-driven circuits using a comparator and logic gates together with the charge amplifier and analog readout circuits for the case of testing an event-driven type of X-ray energy spectrum measurements [20]. In the measurement results throughout this paper, however, the function of event detection and event-driven measurements is not used because the scope of this paper is to characterize the basic pixel performance. 

#### 4.1.1. Linearity and Conversion Gain 

**Figure 13:**
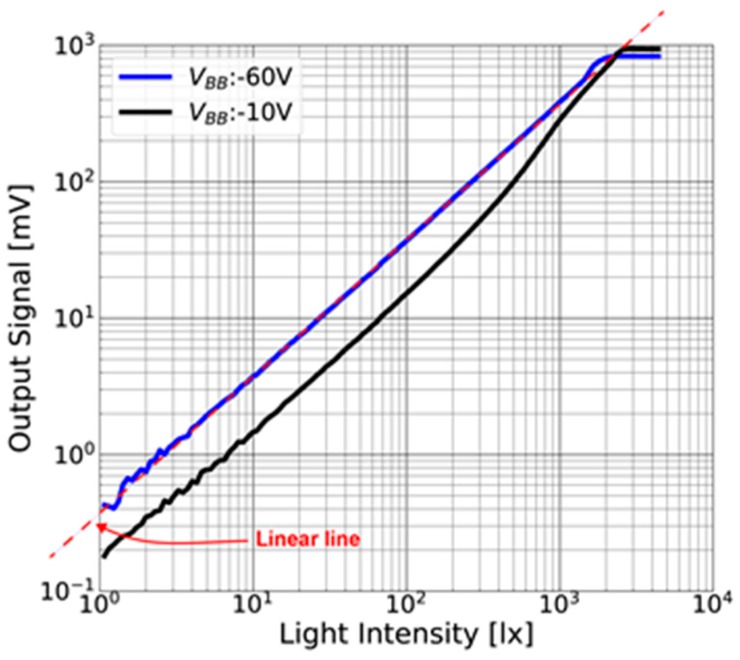
Linearity of the pixel output to light intensity.

Figure 13 shows the linearity measurement of the pixel for the two backside biases (V_BB_) of −10 V and −60 V. A white light generated and intensity-scanned by an LB-8611A precision lighting box (Kyoritsu, Tokyo, Japan) is used for the linearity measurements. The light is illuminated from the backside of the chip. With the thick (200 µm) high-resistivity (25 kΩcm) substrate, the substrate is fully depleted by |V_BB_|of higher than 14.4 V. For V_BB_ of −10 V, the linearity and sensitivity is poor because of incomplete depletion of the substrate. With a fully-depleted biasing of V_BB_ = −60 V, a good linearity is obtained in the output range up to 0.6 V. 

**Figure 14:**
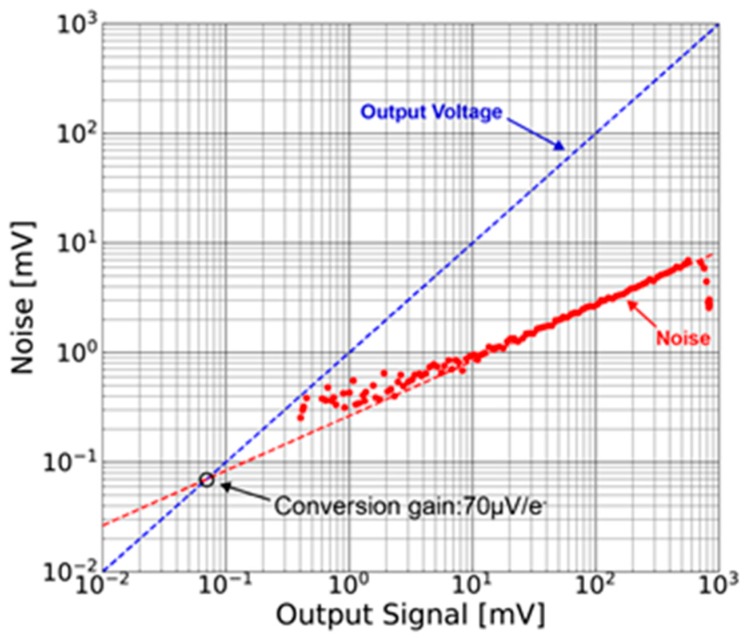
Noise versus signal for conversion gain measurement.

Figure 14 shows the noise as a function of signal amplitude for the measurement of conversion gain. Photon shot noise is used for the measurement of the conversion gain [21]. From the cross point of signal voltage and shot noise voltage, the conversion gain is measured to be 70 µV/e^−^. 

#### 4.1.2. Dark Current

**Figure 15:**
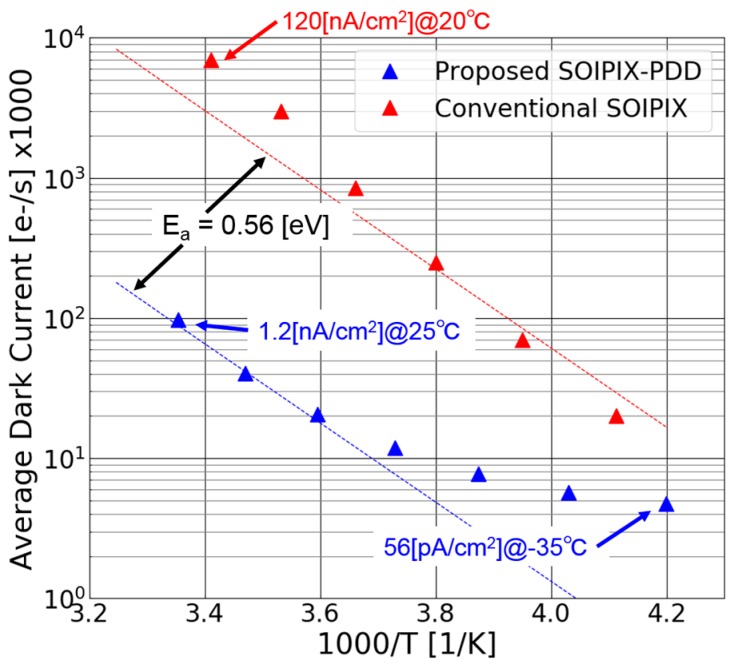
Temperature dependence of dark current.

Figure 15 shows the temperature dependence of dark current of the SOIPIX-PDD. The dark current of the conventional SOI pixel [9] is also shown for comparison. For the comparison at ambient temperature of around 25 °C, the SOIPIX-PDD has 100 times smaller dark current density than that of the conventional SOI pixel. This shows the effectiveness of the pinned depleted diode structure using neutralized BPW layer created just under the BOX to fill the surface with holes for the reduction of the dark current. For the temperature range of higher than 5 °C, or smaller than 3.6/1000 [1/K] in the Arrhenius plot, it follows the line that the activation energy of dark current is 0.56 eV, the half of band gap (= 1.12 eV) of silicon, indicating that the dominant dark current component is still due to SRH generation current of the detector. For the temperature range of smaller than 5 °C, or larger than 3.6/1000 [1/K] in the Arrhenius plot, the temperature dependency tends to saturate and it takes 56 [pA/cm^2^] at −35 °C. The reason for this limitation of the dark current reduction at low temperature is not clear at the moment, but the possible reason is a leakage current of the p-MOS transistor used for resetting the sensing node. This is because the trap-assisted band-to-band tunneling, which is often a major mechanism of leakage current of MOS transistor, has small temperature dependence. 

**Figure 16:**
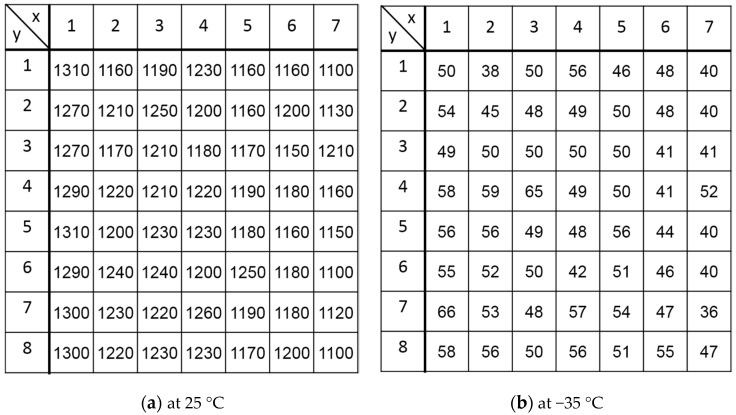
Pixel-to-pixel deviation of dark current.

Figure 16 shows a map of distribution of the dark current measured at 25 °C and −35 °C. The mean and standard deviation are 1200 [pA/cm^2^] and 51.6 [pA/cm^2^] (= 4.3% of mean value) at 25 °C and and 56 [pA/cm^2^] and 6.3 [pA/cm^2^] (= 12.6% of mean value) at −35 °C.

#### 4.1.3 Readout Noise 

**Figure 17:**
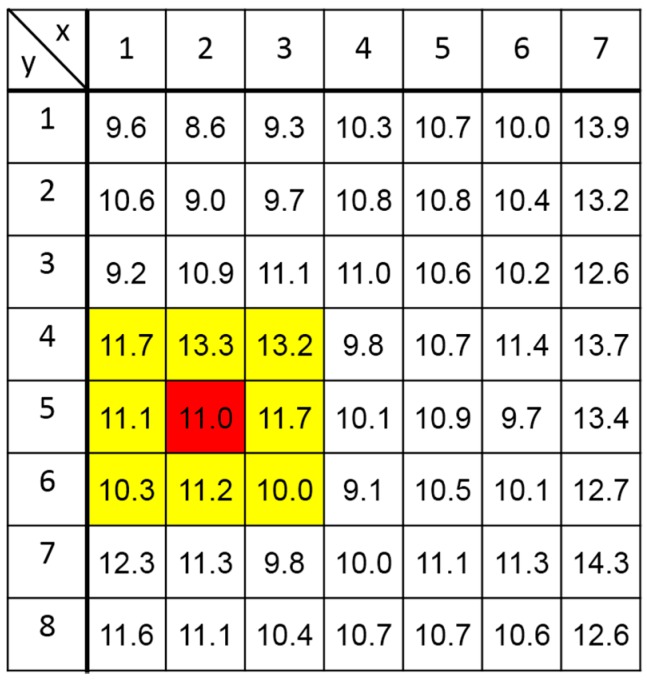
Map of input referred noise [e^−^_rms_] of an 8 × 7 pixel array with cooling at −35 °C. Highlighted 3 × 3 pixel array is used for X-ray energy spectrum measurement. A pixel (x = 2, y = 5) shown by red-colored box is used for the measurement of single pixel events. Adjacent 8 pixels shown by yellow-colored boxes are used for eliminating events of charge splitting to plural of pixels.

Figure 17 shows measured input-referred readout noise of all the 8 × 7 pixels. The average value of the noise is 11.0 e^−^_rms_ and the minimum and maximum noises are 8.6 e^–^_rms_ and 14.3 e^−^_rms_, respectively. Compared with the conventional SOI pixel whose readout noise is 35 e^−^_rms_ [9], the readout noise of the SOIPIX-PDD is reduced to one-third of that. The readout noise calculated by Equation (4) and designed parameters (*C_FB_* = 1.5 [fF], *T_CDS_* = 1 [ms], *τ_CSA_* = 0.2 µs, CS = 240 [fF])) is 4.5 e^−^_rms_ at 107 µV/e^−^. With the measured conversion gain in Figure 14, i.e., 70 µV/e^−^, the readout noise using Equation (4) is calculated to be 5.8 e^−^_rms_ if CFB = 2.3 [fF]. In any way, measured readout noise is bigger than calculated noise. The reason for increased readout noise is considered to be an increase in *C_D_*, an increase in C_FB_ due to parasitic capacitance, and coupling of power supply noise through power and ground lines and substrates. 

### 4.2. X-ray Eergy Sectrum

**Figure 18:**
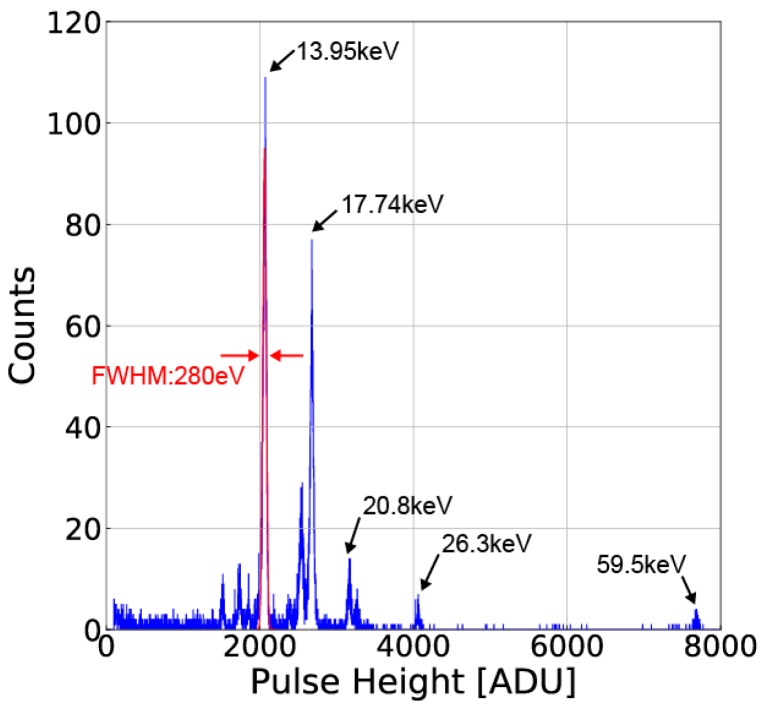
Measured X-ray spectra of ^241^Am as single-pixel events using the SOIPIX-PDD. 1 ADU is 122 μV/e^−^ (2 V/14 bit).

To evaluate the pixel performance for X-ray energy spectrum measurement, a particular pixel (x = 2, y = 5) of the 8 × 7 pixel array is used for the measurement of single pixel events. The adjacent eight pixels are used for eliminating events of charge splitting to plural of pixels. To do this, if the pixel for spectrum measurement has an event by checking whether if the signal is larger than the event threshold, and only if the signals of all adjacent eight pixels are smaller than a given threshold, i.e., the split threshold, the event is counted as the measured energy level in the spectrum. Figure 18 shows the ^241^Am X-ray spectra of single pixel events obtained with the SOIPIX-PDD chip after the data reduction and analyses given in [20]. Since the measurement system uses a 14-bit A/D converter with the analog range of 2 V, 1 ADU corresponds to a bin of 122 μV and an energy bin of 6.54 eV with the conversion gain of 70 μV/e^−^ and ω (= energy required to liberate one electron-hole pair) of 3.65 eV/e^−^. Figure 18. In this measurement, the X-ray event only at the pixel (x = 2, y = 5) and adjacent 8 pixels shown by yellow-colored zone in Figure 17 is considered for the evaluation of single pixel events. The energy resolution of the SOIPIX-PDD is 280 eV (2.01%) in FWHM at 13.95 keV. In this measurement, the event threshold, split threshold and energy bin of the spectrum are set to 10 ADU (= 65 eV), 10 ADU (= 65 eV) and 1.3 ADU (= 8.5 eV), respectively. In the conventional SOIPIX, as given in [20], the FWHM of 1500 eV (10.8%) at 13.95 keV of the ^241^Am was obtained, indicating the effectiveness of the SOIPIX-PDD for improving the energy resolution by a factor of more than 5. Another good effect in the SOIPIX-PDD when compared with the conventional SOI pixels is the very-small tailing structures of the energy spectrum to lower-energy side which is possibly caused by the signal charge loss in the sensor layers [9]. The result of Figure 18 shows the proposed detector has high charge collection efficiency or small signal charge loss thanks to the employment of the pinned depleted diode structure. The theoretical limit of energy resolution (FWHM) of a detector can be found using:

(7) $$\Delta E\left(eV\right)=2.354\omega \sqrt{\frac{FE}{\omega }+\sigma^{2}}$$

where *F* is the Fano factor (0.11 for silicon), *E* is the energy of the incident X-ray photon, and σ is measured readout noise. With the measured noise of the pixel (x = 2, y = 5), i.e., 11.0 e^−^, the energy resolution using Equation (7) is calculated to be 200 eV (1.43%) at E = 13.95 keV. Therefore, there is a factor for further improvement of the energy resolution other than the readout noise. In the measurement of Figure 18, because the radiation source irradiates from the surface side of the SOI pixel, there still exists a possibility of signal charge loss due to the recombination in the neutral BPW layer of the SOIPIX-PDD detector. 

**Figure 19:**
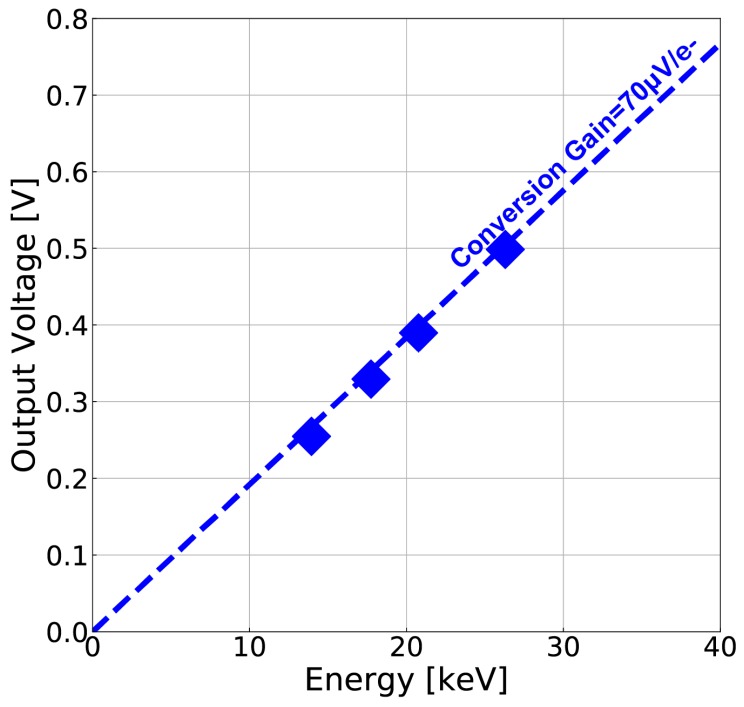
Confirmation of conversion gain with the measured X-ray spectra of ^241^Am.

Figure 19 demonstrates the conversion gain of 70 µV/e^−^ obtained by shot noise measurement to agree with the measured signal voltages to the ^241^Am’s characteristic X-ray lines of 13.95, 17.74, 20.8 and 26.3 keV. In Figure 18, a spectral peak probably due to the characteristic X-ray line of 59.5 keV is observed at the pulse height of 7700 ADU. This pulse height corresponds to 0.94 V as the output signal voltage, while the implemented pixel has the output voltage linearity up to 0.6 V as shown in Figure 13. The characteristic X-ray line of 59.5 keV cannot be exactly measured because the pulse height is outside of the linear range of the designed detector. Therefore the data point for 59.5 keV is not included in Figure 19. 

**Figure 20:**
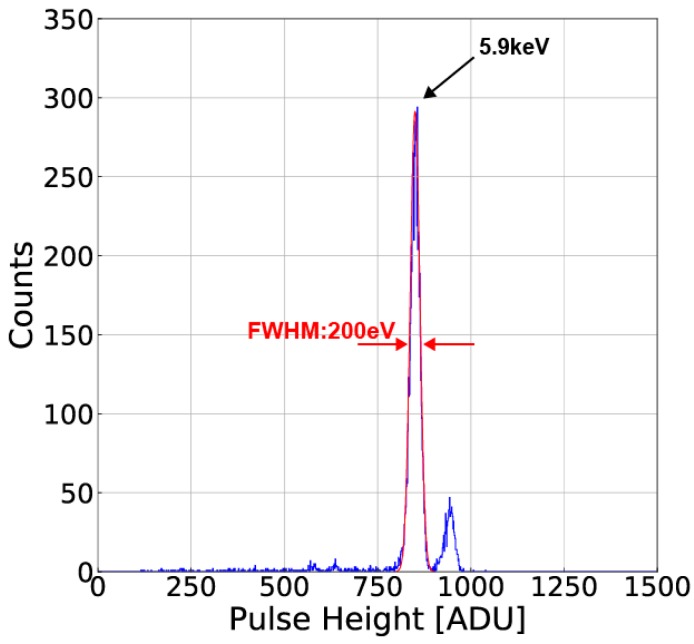
Measured X-ray spectra of ^55^Fe as single-pixel events using the SOIPIX-PDD.

Figure 20 shows the ^55^Fe X-ray spectra of single pixel events using the pixel(x = 2, y = 2) of the SOIPIX-PDD detector of a different chip from that used for measurement of Figure 13, Figure 14, Figure 15, Figure 16, Figure 17, Figure 18 and Figure 19. The bias voltage of V_BB_ and V_BB2_ are set at −60 V, and −2.7 V, respectively. A very good energy resolution (FWHM) of 200 eV (3.6%) at 5.9 keV and very small tailing are obtained. The Mn-K (5.9 keV) and Mn-K (6.4 keV) lines are definitely discriminated. 

### 4.3. Performance Comparison

Table 2 shows a comparison of the conventional SOIPIX (XRPIX series) and SOIPIX-PDD. Using SOIPIX-PDD, the readout noise and dark current are significantly reduced and the resulting X-ray spectroscopic performance greatly improved when compared with the conventional SOIPIX detectors. Though it is not shown in the Table 2, characteristic X-ray spectral line with very small tailing because of high charge collection efficiency is another advantage of the SOIPIX-PDD.

## 5. Conclusions

A novel SOI pixel detector using a pinned depleted diode structure (SOIPIX-PDD) has been presented in this paper. The SOIPIX-PDD realizes a low readout noise due to small charge sensing node capacitance, low dark current due to a pinned Si surface at Si-SiO_2_ interface of the detector under the BOX layer and high charge collection efficiency with the buried channel for carrier collection. The implemented chip demonstrates that the SOIPIX-PDD pixels has a high-conversion gain of 70 µV/e^−^, low noise of 11.0 e^−^_rms_, low dark current of 56 pA/cm^2^ at −35 °C, and good energy resolution in the measured characteristic X-ray lines, e.g., 200eV(FWHM) at 5.9 keV and 280 eV (FWHM) at 13.95 keV. 

## Figures and Tables

**Table 1:** SOIPIX Process Technology.

Process	0.20 µm FD-SOI CMOS Technology with Substrate-Detector Process
Substrate thickness	200 µm
Wafer type	FZ-p (Floating Zone, p-type)
Substrate Resistivity	>25 kΩcm

**Table 2:** Comparison with the conventional SOIPIX (XRPIX series) and SOIPIX-PDD.

SOIPIX Type	XRPIX1 [20]	XRPIX2b-A [9]	XRPIX3b-CSA [9]	SOIPIX-PDD
Conversion gain	3.56	7.0	17.8	70
Readout noise	129 e^−^_rms_	68 e^−^_rms_	35 e^−^_rms_	11.0 e^−^_rms_
Dark current	N. A.	N. A.	120 nA/cm^2^@25 °C	1.2 nA/cm^2^@20 °C
Energy resolution (FWHM@5.9 keV)	N. A.	N. A.	320 eV (5.4%)	200 eV (3.6%)
Energy resolution (FWHM@13.95 keV)	1500 eV (10.8%)	(~1500 eV)*	N. A.	280 eV (2.0%)

* Numerical value of energy resolution is not reported, but it is estimated from the graph of X-ray energy spectra for ^241^Am given in [9].
